# Illicit Drug Use in Canada and Implications for Suicidal Behaviors, and Household Food Insecurity: Findings from a Large, Nationally Representative Survey

**DOI:** 10.3390/ijerph18126425

**Published:** 2021-06-14

**Authors:** Nicola Luigi Bragazzi, Dan Beamish, Jude Dzevela Kong, Jianhong Wu

**Affiliations:** 1 Centre for Disease Modelling, Department of Mathematics and Statistics, York University, Toronto, ON M3J 1P3, Canada; jdkong@yorku.ca (J.D.K.); wujhhida@gmail.com (J.W.); 2 Department of Emergency Medicine, University of Ottawa, Ottawa, ON K1Y 4E9, Canada; dan.beamish@gmail.com; 3 Department of Emergency Medicine, Pembroke Regional Hospital, Pembroke, ON K8A 1G8, Canada

**Keywords:** illicit drug use, suicidal behavior, household food insecurity, Canada

## Abstract

*Background and Aims*: Illicit drug use is an ongoing health and social issue in Canada. This study aimed to investigate the prevalence of illicit drug use and its implications for suicidal behaviors, and household food insecurity in Canada. *Design*: Cross-sectional population survey. *Setting*: Canada, using the 2015–2016 Canadian Community Health Survey, a nationally representative sample selected by stratified multi-stage probability sampling. *Participants*: A total of 106,850 respondents aged ≥ 12 years who had completed information on illicit drug use. *Measurements*: Illicit drug use was assessed through a series of questions about illicit drug use methods. Respondents who reported lifetime illicit drug use but no past-year use were considered to have prior illicit drug use. In this survey, illicit drug use included cannabis use. *Findings*: Overall, the prevalence of lifetime, past-year, and prior illicit drug use was 33.2% (9.8 million), 10.4% (3.1 million), and 22.7% (6.7 million), respectively. In models adjusting for sociodemographic covariates, prior illicit drug use was significantly associated with increased odds of past-year suicidal ideation (adjusted odds ratio [AOR] 1.21, 95% CI 1.04–1.40), and plans (1.48, 1.15–1.91), and past-year household food insecurity (1.27, 1.14–1.41), and the odds were much higher among prior injecting drug users than prior non-injecting drug users. No significant correlation was found between prior illicit drug use and past-year suicidal attempts, but there was a strong association between past-year illicit drug use and past-year suicidal attempts. *Conclusions*: Our findings suggest that even after people have stopped taking illicit drugs, prior illicit drug use, especially for prior injecting drug use, continues to be associated with increased risks of subsequent suicidal ideation, and plans, and household food insecurity.

## 1. Introduction

Illicit drugs are substances which non-medical use is prohibited by international drug control treaties [1]. They commonly include cannabis, opioids, cocaine, amphetamine-type substances, and ecstasy-group substances [2]. Worldwide, an estimated 275 million people aged 15–64 years used an illicit drug at least once during 2016, and about 31 million of these illicit drug users suffered from drug use disorders [3].

Canada is one of the countries with the highest prevalence of illicit drug use [4]. Due to the ineffectiveness of previous legal prohibition, the Canadian federal government decriminalized the use of recreational cannabis among adults in 2018. Following this, there has been a growing campaign including medical officers of health that have called for the decriminalization of all illicit drugs [5,6]. However, to our knowledge, available evidence on the national prevalence and methods of illicit drug use and its implications in Canada are limited.

Recently, a growing body of literature has linked illicit drug use to suicidal behaviors, and household food insecurity [7,8,9,10,11,12,13,14,15]. Illicit drug use represents a multi-faceted, complex phenomenon, characterized by a subtle, non-linear interplay between several variables including family relationships, difficult experiences, and strategies for coping with stress, among others. Although most of published studies have assumed that illicit drug use contributes to suicidal behaviors, and household food insecurity, longitudinal studies examining sequential associations between illicit drug use and suicidal behaviors, and household food insecurity are scarce, which prevents the attribution of public health detriments to illicit drug use. Additionally, because illicit drug use is unlikely to be completely eliminated in Canada, identifying sub-population of illicit drug users at higher risks for suicidal behaviors, and household food insecurity would critically inform public policy.

In this study, we used a nationally representative Canadian sample to: (1) estimate the prevalence and sociodemographic correlates of illicit drug use; (2) investigate the patterns of illicit drug use methods; (3) examine the cross-sectional and longitudinal associations of illicit drug use with suicidal behaviors, and household food insecurity; and (4) identify whether injecting drug users are at higher risks for suicidal behaviors, and household food insecurity than non-injecting drug users.

## 2. Methods

### 2.1. Data and Sample

We analyzed data from the 2015–16 Canadian Community Health Survey. Details about this survey can be found elsewhere [16]. Briefly, 110,100 respondents aged ≥ 12 years living in the 10 provinces and three territories were selected by Statistics Canada to estimate the general health of the Canadian population. Residents living on Indian Reserves, Crown Lands, institutions, certain remote regions, and full-time members of the Canadian Forces were excluded. Statistics Canada estimated that approximately 98% of the Canadian population aged ≥ 12 years was covered by the survey. In this study, we limited the sample to respondents who had not missing data for illicit drug use. All respondents provided written informed consent, and ethical approval was granted by the relevant policy committees at Statistics Canada.

### 2.2. Measures

Illicit drug use was assessed through a series of questions about illicit drug use methods: (1) have you ever smoked illicit drugs, and whether this experience was in the past 12 months; (2) have you ever taken illicit drugs orally (swallowed illicit drugs), and whether this experience was in the past 12 months; (3) have you ever snorted or sniffed illicit drugs, and whether this experience was in the past 12 months; and (4) have you ever used a needle to inject or be injected with a drug not prescribed by a doctor, and whether this experience was in the past 12 months. In this survey, illicit drug use included cannabis use as the survey was done prior to the legalization of recreational use of cannabis in 2018. Lifetime illicit drug use was defined as present if respondents answered “yes” to any experience that has ever happened. Past-year illicit drug use was defined as present if respondents answered “yes” to any experience that happened in the past 12 months. Prior illicit drug use was defined as present if respondents had lifetime illicit drug use but no past-year use.

Past-year suicidal behaviors, including past-year suicidal ideation, plans, and attempts, were determined by asking whether they had seriously contemplated suicide in the past 12 months, whether they had made a plan to seriously attempt suicide in the past 12 months, and whether they had seriously attempted suicide in the past 12 months. Each suicidal behavior was defined as present if respondents answered “yes” to the corresponding question.

Past-year household food security was measured using an 18-item scale [17], and was categorized as food secure and food insecure according to Health Canada’s coding method [18]. The measurement focused on the financial ability of respondents’ households, including both adults and children, to access adequate food [18].

Sociodemographic variables were selected according to previous studies [19,20,21], and included age, sex, race/ethnicity, country of birth, marital status, education level, household income, and province/territory of residence.

### 2.3. Statistical Analyses

All analyses were performed using Stata 15.0 (StataCorp LLC, College Station, TX, USA) and R 3.5.1 (R Foundation for Statistical Computing, Vienna, Austria). Sampling weights were applied to ensure that the estimates reflected general population covered by the Canadian Community Health Survey [22]. Bootstrapping with 1000 replicates was used to account for the complex survey design. First, prevalence estimates and 95% confidence intervals (CI) were computed for illicit drug use, and illicit drug use methods. Second, we constructed multivariable logistic regression models to test the sociodemographic correlates of illicit drug use. Third, we used marginal standardization method [23] to compute age- and sex-adjusted percentages with 95% CI for past-year suicidal behaviors, and past-year household food insecurity across different populations.

Fourth, we reconstructed a series of multivariable logistic regression models adjusted for all sociodemographic variables to examine the associations of lifetime, past-year, and prior illicit drug use with past-year suicidal behaviors, and past-year household food insecurity; and to determine whether the associations differed between injecting drug users and non-injecting drug users. In these models, illicit drug use was firstly classified as “no illicit drug use” and “any illicit drug use”, and then classified as “no illicit drug use”, “non-injecting drug use”, and “injecting drug use”. Finally, we computed population attributable fractions (PAF) through a user-written Stata command “punaf” to estimate the proportion of adverse outcomes that might be decreased among prior illicit drug users if they did not have prior illicit drug use [24].

## 3. Results

Of the 110,100 respondents, 3250 were excluded because of missing data on illicit drug use; leaving 106,850 respondents in our analysis. These respondents were weighted to represent 29,566,000 Canadians aged ≥ 12 years. Overall, 33.2% (95% CI 32.6–33.7) of the respondents reported lifetime illicit drug use, 10.4% (95% CI 10.1–10.8) reported past-year illicit drug use, and 22.7% (95% CI 22.3–23.2) reported prior illicit drug use. These percentages represented approximately 9.8 million, 3.1 million, and 6.7 million Canadians, respectively.

**Figure 1:**
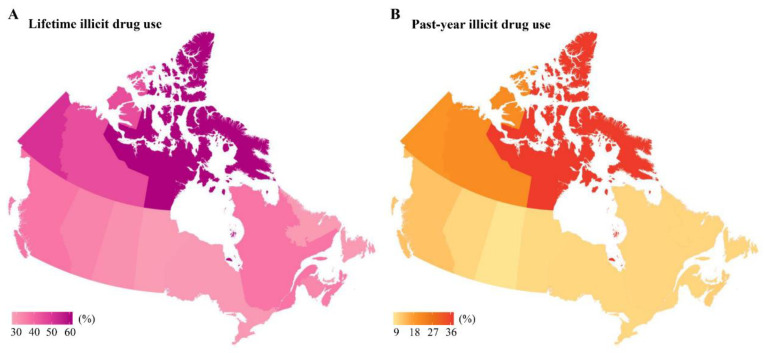
Prevalence of lifetime (**A**), and past-year (**B**) illicit drug use by province/territory in Canada.

Figure 1 and Table 1 show that there were significant variations in illicit drug use across provinces and territories. The prevalence of lifetime illicit drug use varied between 28.4% (95% CI 25.9–31.0) in Prince Edward Island and 61.1% (95% CI 54.0–67.9) in Nunavut, and the prevalence of past-year illicit drug use varied between 8.5% (95% CI 7.4–9.7) in Saskatchewan and 36.8% (95% CI 32.0–42.0) in Nunavut. Table 2 depicts other sociodemographic correlates of lifetime and past-year illicit drug use. Respondents aged 30 to 39 years were most likely to report lifetime illicit drug use, and respondents aged 20 to 29 years were most likely to have past-year illicit drug use. The odds of both lifetime and past-year illicit drug use were greater in men, in those who identify as Aboriginal, in respondents born in Canada, in respondents who are separated/divorced/widowed or never married, and in respondents who report a household income of ≤29 999 CAD$. Interestingly, when compared with those who are less than secondary school graduation, respondents with post-secondary graduation had significantly higher lifetime illicit drug use, but no significantly higher past-year illicit drug use.

**Figure 2:**
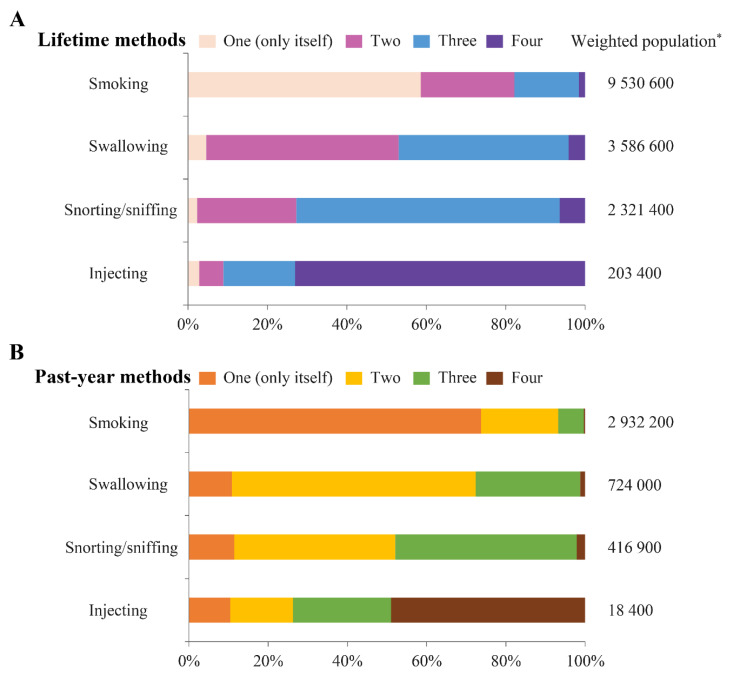
Patterns of lifetime (**A**), and past-year (**B**) illicit drug use methods. * Weighted numbers were rounded to base 100 for confidentiality purposes according to Statistics Canada data release policies.

With respect to illicit drug use methods, smoking was the most common, followed by swallowing, snorting/sniffing, and injecting. The lifetime prevalence of smoking, swallowing, snorting/sniffing, and injecting drug use was 32.2% (95% CI 31.7–32.8), 12.1% (95% CI 11.8–12.5), 7.9% (95% CI 7.6–8.1), and 0.7% (95% CI 0.6–0.8), respectively. The past-year prevalence of smoking, swallowing, snorting/sniffing, and injecting drug use was 9.9% (95% CI 9.6–10.2), 2.4% (95% CI 2.3–2.6), 1.4% (95% CI 1.3–1.5), and 0.06% (95% CI 0.04–0.09), respectively. Notably, 73.1% (95% CI 68.4–77.3) of lifetime injecting drug users also used all other three methods to take illicit drugs in their lifetime, and 49.0% (95% CI 32.0–66.2) of past-year injecting drug users also used all other three methods to take illicit drugs in the past 12 months (Figure 2 and Table 3).

Table 4 shows the association of lifetime and past-year illicit drug use with past-year suicidal behaviors, and past-year household food insecurity. In models adjusting for sociodemographic covariates, lifetime illicit drug use was significantly associated with increased odds of having past-year suicidal ideation, plans, and attempts, and past-year household food insecurity. After further dividing lifetime illicit drug use into lifetime injecting drug use and lifetime non-injecting drug use, we found that the odds of having past-year suicidal ideation, plans, and attempts, and past-year household food insecurity were much higher among lifetime injecting drug users than lifetime non-injecting drug users. Similar results were obtained with past-year illicit drug use.

All models were adjusted for age, sex, race/ethnicity, country of birth, marital status, education level, household income, and province/territory of residence.

For household food insecurity, the survey module was not adopted by Newfoundland and Labrador, Ontario, and Yukon; thus, only 67,550 respondents were included into the analyses.

Finally, we explored the association of prior illicit drug use with past-year suicidal behaviors, and past-year household food insecurity (Table 4 and Table 5). In models adjusting for sociodemographic covariates, prior illicit drug use was significantly associated with increased odds of past-year suicidal ideation, and plans, and past-year household food insecurity, and the odds were similarly higher among prior injecting drug users than prior non-injecting drug users. However, no significant correlation was found between prior illicit drug use and past-year suicidal attempts. The PAF estimates suggested that a substantial proportion of past-year suicidal ideation, and plans, and past-year household food insecurity might be decreased among prior illicit drug users if they did not have prior illicit drug use (Table 5).

Also in this case, all models were adjusted for age, sex, race/ethnicity, country of birth, marital status, education level, household income, and province/territory of residence.

For household food insecurity, the survey module was not adopted by Newfoundland and Labrador, Ontario, and Yukon; thus, only 67,550 respondents were included into the analyses.

## 4. Discussion

Our findings indicate that illicit drugs were commonly used among Canadians aged ≥ 12 years with 33.2% (9.8 million) reporting lifetime use, 10.4% (3.1 million) reporting past-year use, and 22.7% (6.7 million) reporting prior use. There was substantial interprovincial or inter-territorial variability in the prevalence of illicit drug use in Canada. Nunavut, Northwest Territories, and Yukon had an obviously higher prevalence of both lifetime and past-year illicit drug use, probably due to the harsh climate, suggesting a more urgent policy attention in these territories. Moreover, our findings show that prior illicit drug use, especially for prior injecting drug use, was significantly associated with increased odds of past-year suicidal ideation, and plans, and past-year household food insecurity.

This study is a valuable complement to the Canadian Tobacco, Alcohol and Drugs Survey [25] from another perspective—illicit drug use methods, with the inclusion of all Canadian provinces/territories and a much larger sample size. From the distribution of lifetime illicit drug use by age groups, we can see that illicit drug use was rising in recent decades. Among those aged ≥70 years, only a relatively small proportion reported lifetime illicit drug use. Besides, we should note that even teenagers aged 12 to 19 years reported a high prevalence of illicit drug use. Taking account of the striking disability-adjusted life years attributable to illicit drug use among youth [26], efficient strategies are truly urgent for this population. The association of being men, identifying as Aboriginal, not being married/common-law, and having a low household income with illicit drug use was unsurprising, as findings from numerous past studies were consistent with this [8,27,28]. Immigrants appeared to be less likely to have illicit drug use. Further research is needed to confirm whether cultures or religious beliefs accepted during childhood have long-term protective influences on illicit drug use. The finding regarding the association between education level and illicit drug use is noteworthy. It seems that people with post-secondary graduation were more likely to quit illicit drug use.

Degenhardt et al. [29] reported that an estimated 308,000 (1.22%) Canadians aged 15 to 64 years were injecting drug users in 2004. However, the prevalence was derived from 21 subregional studies using an indirect method, and the data seems outdated [29]. To our knowledge, the present study is the first to provide direct nationwide estimates of the prevalence of injecting drug use as well as the prevalence of smoking, swallowing, and snorting/sniffing drug use in Canada. Additionally, our study showed that a high proportion of injecting drug users also used all other three methods to take illicit drugs, implying that injecting drug users were likely to be polydrug abusers or have more significant drug use disorders.

The cross-sectional association between illicit drug use and suicidal behaviors has been consistently documented [7,8,9,10,11], yet longitudinal studies have yielded conflicting results. For example, using four waves of data from 3342 respondents, Zhang et al. [30] found that cannabis and other illicit drug use did not increase risk of suicidal ideation. In contrast, a 2019 systematic review [31] concluded that cannabis use during adolescence was associated with subsequent suicidal ideation, and attempts within adolescence or in young adulthood when pooling five studies; however, none of the five studies have investigated the cannabis use status at the end of the studies, which may influence the results.

In this study, we found that prior illicit drug use (no past-year use) had a persistent association with increased odds of past-year suicidal ideation, and plans, implying that illicit drug use has long-term impacts on suicidality. On the other hand, compared with the strong association between past-year illicit drug use and past-year suicidal behaviors, the odds of having past-year suicidal ideation, and plans have been attenuated for prior illicit drug use, and no correlation was found between prior illicit drug use and past-year suicidal attempts. A potential explanation is that the impacts of illicit drug use on suicidal behaviors, especially on suicidal attempts, are time-dependent. Furthermore, very few previous studies have explored whether association between illicit drug use and suicidal behaviors differed for different methods of illicit drug use [32]. In this study, we concluded that injecting drug users were at higher risks for suicidal behaviors than non-injecting drug users.

Our results also agree with previous studies that have shown cross-sectional associations between illicit drug use and household food insecurity [12,13,14,15]. However, to date and to our knowledge, there has been no study examining the longitudinal impacts of illicit drug use on household food insecurity. In this study, we firstly concluded that prior illicit drug use, especially prior injecting drug use, was significantly associated with subsequent household food insecurity. Given that household food insecurity greatly increases child malnutrition and health risks [33,34], policy efforts on illicit drug users should also focus on their households, especially for the children.

The strengths of our study include the large and nationally representative sample of Canadians, detailed information on illicit drug use methods, and a longitudinal design. However, several limitations should be noted. First, at the time of survey, all illicit drugs, including recreational cannabis, were legally prohibited in Canada. Although respondents had been informed of the strict confidentiality policy of this survey [16], the true prevalence of illicit drug use was likely underestimated. Second, our ability to make causal inferences is limited by the observational nature of the study. However, both the longitudinal associations identified in our study and the finding that injecting drug users had higher odds of adverse consequences than non-injecting drug users support the possibility of a causal relation. Third, our interpretation is hindered by lack of information on initiation, types, and duration of illicit drug use. Nevertheless, our study has provided novel and reliable evidence on the implications of illicit drug use, which is very important for public policy decisions. Further studies are needed to address these shortcomings.

## 5. Conclusions

Our study suggests that illicit drug use is very common in Canada. Even after people have stopped taking illicit drugs, prior illicit drug use, especially prior injecting drug use, continues to be associated with increased risks of subsequent suicidal ideation, and plans, and household food insecurity. From a public health perspective, interventions targeting illicit drug users, particularly injecting drug users, are very necessary in Canada, and the efforts should not only focus on the users but also their households.

## Figures and Tables

**Table 1:** Prevalence of lifetime, and past-year illicit drug use by province/territory in Canada.

Geographic Location	n †	Lifetime Illicit Drug Use Percentage (95% CI) AOR (95% CI) ‡ *p* Value			Past-Year Illicit Drug Use Percentage (95% CI) AOR (95% CI) ‡ *p* Value		
Province/Territory of Residence							
Saskatchewan	4550	31.8% (29.9–33.7)	1 (ref)		8.5% (7.4–9.7)	1 (ref)	
Manitoba	5300	29.0% (27.2–30.8)	1.00 (0.87–1.14)	0.9578	9.9% (8.7–11.1)	1.30 (1.05–1.62)	0.0179
Newfoundland and Labrador	3100	29.5% (27.3–31.8)	0.84 (0.72–0.97)	0.0196	10.3% (9.0–11.9)	1.31 (1.03–1.67)	0.0260
Quebec	23,100	36.9% (35.9–37.9)	1.40 (1.26–1.56)	<0.0001	10.2% (9.5–10.8)	1.38 (1.15–1.64)	0.0005
New Brunswick	3200	33.6% (31.5–35.7)	1.03 (0.90–1.19)	0.6348	10.3% (8.9–11.9)	1.33 (1.05–1.69)	0.0178
Alberta	12,800	34.0% (32.6–35.5)	1.27 (1.12–1.43)	0.0001	10.3% (9.5–11.2)	1.42 (1.18–1.72)	0.0003
Prince Edward Island	1800	28.4% (25.9–31.0)	0.86 (0.72–1.02)	0.0769	10.2% (8.5–12.1)	1.33 (1.02–1.75)	0.0375
Nova Scotia	4600	35.4% (33.6–37.2)	1.18 (1.03–1.35)	0.0139	11.2% (10.0–12.5)	1.47 (1.19–1.82)	0.0004
Ontario	31,650	29.7% (28.8–30.6)	1.29 (1.16–1.44)	<0.0001	10.0% (9.4–10.5)	1.61 (1.35–1.91)	<0.0001
British Columbia	13,900	37.0% (35.6–38.4)	1.93 (1.71–2.17)	<0.0001	12.6% (11.7–13.5)	2.16 (1.79–2.60)	<0.0001
Yukon	950	51.4% (46.9–55.8)	2.32 (1.86–2.90)	<0.0001	19.3% (16.5–22.5)	2.46 (1.84–3.28)	<0.0001
Northwest Territories	1000	45.2% (41.0–49.4)	1.28 (1.02–1.61)	0.0364	20.9% (17.3–25.0)	2.07 (1.50–2.87)	<0.0001
Nunavut	850	61.1% (54.0–67.9)	2.31 (1.65–3.24)	<0.0001	36.8% (32.0–42.0)	3.54 (2.58–4.85)	<0.0001

Abbreviations: AOR, adjusted odds ratio; CI, confidence interval. † Unweighted numbers were reported. They were rounded to base 50 for confidentiality purposes according to Statistics Canada data release policies. Percentages were based on weighted numbers. ‡ Adjusted for all covariates listed in Table 1.

**Table 2:** Prevalence of lifetime, and past-year illicit drug use in Canada.

Socio-Demographic Characteristics	n †	Lifetime Illicit Drug Use Percentage (95% CI) AOR (95% CI) ‡ *p* Value			Past-Year Illicit Drug Use Percentage (95% CI) AOR (95% CI) ‡ *p* Value		
Overall	106,850	33.2% (32.6–33.7)		·	10.4% (10.1–10.8)		
Age, years							
12 to 19	10,750	13.4% (12.4–14.4)	1 (ref)	··	10.1% (9.2–11.0)	1 (ref)	
20 to 29	11,500	44.0% (42.4–45.6)	5.49 (4.81–6.25)	<0.0001	23.5% (22.1–24.8)	3.21 (2.73–3.78)	<0.0001
30 to 39	15,050	46.6% (45.3–47.9)	7.01 (6.13–8.01)	<0.0001	15.9% (15.0–16.9)	2.74 (2.31–3.25)	<0.0001
40 to 49	14,200	35.4% (34.1–36.7)	4.22 (3.69–4.83)	<0.0001	8.0% (7.4–8.7)	1.25 (1.04–1.50)	0.0193
50 to 59	18,000	39.4% (38.2–40.6)	4.35 (3.81–4.96)	<0.0001	7.3% (6.8–8.0)	0.98 (0.82–1.17)	0.8184
60 to 69	19,150	29.1% (28.0–30.1)	2.59 (2.26–2.96)	<0.0001	4.1% (3.7–4.6)	0.52 (0.43–0.63)	<0.0001
≥70	18,150	7.9% (7.3–8.5)	0.53 (0.46–0.61)	<0.0001	1.0% (0.8–1.3)	0.11 (0.08–0.15)	<0.0001
Sex							
Women	57,750	27.6% (27.0–28.2)	1 (ref)	··	7.4% (7.1–7.8)	1 (ref)	··
Men	49,100	38.9% (38.2–39.7)	1.82 (1.74–1.91)	<0.0001	13.6% (13.0–14.1)	2.04 (1.89–2.20)	<0.0001
Race/Ethnicity							
White	86,150	38.2% (37.7–38.8)	1 (ref)	··	11.3% (11.0–11.7)	1 (ref)	··
Aboriginal	6300	51.1% (49.0–53.3)	1.54 (1.39–1.70)	<0.0001	22.4% (20.5–24.5)	1.60 (1.39–1.83)	<0.0001
Black	1500	16.0% (13.3–19.1)	0.40 (0.32–0.50)	<0.0001	6.4% (4.9–8.4)	0.52 (0.38–0.72)	0.0001
Asian	6650	9.7% (8.5–11.0)	0.21 (0.18–0.25)	<0.0001	4.1% (3.3–5.0)	0.33 (0.25–0.43)	<0.0001
Other/Mixed Ancestry	3750	20.5% (18.7–22.4)	0.52 (0.45–0.59)	<0.0001	8.4% (7.2–9.8)	0.68 (0.56–0.83)	0.0001
Country of birth							
Canada	87,500	39.7% (39.2–40.2)	1 (ref)	··	12.6% (12.3–13.0)	1 (ref)	··
Other than Canada	17,150	14.9% (14.1–15.9)	0.42 (0.38–0.46)	<0.0001	4.3% (3.8–4.9)	0.47 (0.39–0.56)	<0.0001
Marital status							
Married/common-law	54,350	33.3% (32.7–34.0)	1 (ref)	··	7.2% (6.9–7.6)	1 (ref)	··
Separated/divorced/widowed	21,350	29.9% (28.8–31.1)	1.35 (1.25–1.46)	<0.0001	7.5% (6.9–8.2)	1.73 (1.54–1.94)	<0.0001
Never married	30,950	34.2% (33.2–35.1)	1.22 (1.14–1.31)	<0.0001	17.8% (17.1–18.6)	1.83 (1.66–2.01)	<0.0001
Education level							
Less than secondary school grad	23,750	20.4% (19.6–21.3)	1 (ref)	··	8.6% (8.0–9.2)	1 (ref)	··
Secondary school grad	22,700	35.8% (34.7–36.9)	1.39 (1.27–1.52)	<0.0001	13.6% (12.8–14.3)	1.39 (1.22–1.58)	<0.0001
Post-secondary grad	59,250	36.0% (35.3–36.7)	1.39 (1.29–1.50)	<0.0001	9.9% (9.5–10.3)	1.09 (0.97–1.24)	0.1565
Household income, CAD$							
≤29,999	20,450	31.3% (30.1–32.4)	1 (ref)	··	13.2% (12.4–14.1)	1 (ref)	··
30,000 to 49,999	19,000	28.8% (27.7–29.9)	0.81 (0.75–0.87)	<0.0001	10.4% (9.7–11.2)	0.79 (0.70–0.89)	0.0001
50,000 to 79,999	23,000	31.9% (30.8–32.9)	0.82 (0.76–0.88)	<0.0001	10.3% (9.6–10.9)	0.69 (0.61–0.77)	<0.0001
≥80,000	44,400	35.7% (34.9–36.4)	0.81 (0.76–0.87)	<0.0001	9.7% (9.3–10.2)	0.58 (0.53–0.65)	<0.0001

Abbreviations: AOR, adjusted odds ratio; CI, confidence interval. † Unweighted numbers were reported. They were rounded to base 50 for confidentiality purposes according to Statistics Canada data release policies. Percentages were based on weighted numbers. ‡ Adjusted for all other covariates listed in the table and province/territory of residence.

**Table 3:** Patterns of lifetime, and past-year illicit drug use methods.

Illicit Drug Uptake	Weighted Population †	Number of Illicit Drug Use Methods			
		One (Only Itself)	Two	Three	Four
Lifetime Illicit Drug Use Methods					
Smoking	9,530,600	58.6% (57.7–59.5)	23.6% (22.8–24.3)	16.3% (15.6–17.0)	1.6% (1.4–1.8)
Swallowing	3,586,600	4.6% (4.0–5.2)	48.5% (47.0–49.9)	42.8% (41.4–44.3)	4.1% (3.6–4.8)
Snorting/sniffing	2,321,400	2.3% (1.9–2.8)	25.0% (23.4–26.7)	66.3% (64.6–68.0)	6.4% (5.5–7.4)
Injecting	203,400	2.8% (1.8–4.4)	6.1% (4.2–8.7)	18.0% (14.6–21.9)	73.1% (68.4–77.3)
Past-year illicit drug use methods					
Smoking	2,932,200	73.8% (72.4–75.1)	19.4% (18.2–20.7)	6.5% (5.8–7.4)	0.3% (0.2–0.5)
Swallowing	724,000	10.8% (8.9–13.2)	61.6% (58.2–64.8)	26.4% (23.5–29.4)	1.2% (0.7–2.1)
Snorting/sniffing	416,900	11.5% (8.8–14.8)	40.6% (36.2–45.2)	45.8% (41.4–50.2)	2.2% (1.2–3.7)
Injecting	18,400	10.4% (4.8–21.1)	15.8% (7.1–31.4)	24.8% (14.1–39.9)	49.0% (32.0–66.2)

Abbreviations: CI, confidence interval. † Weighted numbers were rounded to base 100 for confidentiality purposes according to Statistics Canada data release policies. Percentages were based on weighted numbers.

**Table 4:** Association of lifetime, and past-year illicit drug use with past-year suicidal behaviors, and past-year household food insecurity.

Association with Outcome	No Illicit Drug Use	Any Illicit Drug Use	*p* Value †	Any Illicit Drug Use Non-Injecting Drug Use	*p* Value †	Injecting Drug Use	*P* Value †
Lifetime illicit drug use							
Suicidal behaviors (n = 106,100) *							
Suicidal ideation							
Percentage (95% CI)	1.5% (1.3–1.6)	4.4% (4.0–4.8)	<0.0001	4.3% (3.9–4.6)	<0.0001	13.3% (9.0–17.7)	<0.0001
AOR (95% CI)	1 (ref)	2.93 (2.53–3.39)	<0.0001	2.84 (2.44–3.29)	<0.0001	7.56 (4.88–11.70)	<0.0001
Suicidal plans							
Percentage (95% CI)	0.4% (0.3–0.4)	1.5% (1.3–1.7)	<0.0001	1.4% (1.2–1.6)	<0.0001	5.6% (3.4–7.8)	<0.0001
AOR (95% CI)	1 (ref)	3.89 (3.07–4.93)	<0.0001	3.75 (2.95–4.78)	<0.0001	10.59 (6.40–17.53)	<0.0001
Suicidal attempts							
Percentage (95% CI)	0.2% (0.1–0.2)	0.8% (0.6–0.9)	<0.0001	0.7% (0.6–0.9)	<0.0001	3.2% (1.3–5.1)	<0.0001
AOR (95% CI)	1 (ref)	3.63 (2.33–5.65)	<0.0001	3.50 (2.23–5.48)	<0.0001	9.68 (4.60–20.39)	<0.0001
Household food insecurity (n = 67,550) *							
Percentage (95% CI)	6.6% (6.3–7.0)	10.3% (9.7–10.9)	<0.0001	10.0% (9.4–10.6)	<0.0001	27.2% (21.3–33.2)	<0.0001
AOR (95% CI)	1 (ref)	1.84 (1.65–2.05)	<0.0001	1.79 (1.61–2.00)	<0.0001	4.21 (3.04–5.84)	<0.0001
Past-year illicit drug use							
Suicidal behaviors (n = 106,100) *							
Suicidal ideation							
Percentage (95% CI)	1.8% (1.7–1.9)	7.6% (6.8–8.5)	<0.0001	7.5% (6.7–8.4)	<0.0001	22.3% (10.5–34.1)	<0.0001
AOR (95% CI)	1 (ref)	3.54 (3.02–4.14)	<0.0001	3.50 (2.99–4.10)	<0.0001	10.37 (5.02–21.43)	<0.0001
Suicidal plans							
Percentage (95% CI)	0.5% (0.4–0.6)	2.5% (2.0–2.9)	<0.0001	2.4% (1.9–2.9)	<0.0001	15.1% (4.1–26.1)	<0.0001
AOR (95% CI)	1 (ref)	3.79 (2.97–4.85)	<0.0001	3.70 (2.89–4.73)	<0.0001	21.56 (8.70–53.45)	<0.0001
Suicidal attempts							
Percentage (95% CI)	0.2% (0.2–0.3)	1.53% (1.10–1.96)	<0.0001	1.49% (1.06–1.91)	<0.0001	10.6% (0.6–20.7)	<0.0001
AOR (95% CI)	1 (ref)	4.93 (3.30–7.37)	<0.0001	4.79 (3.18–7.20)	<0.0001	31.65 (10.40–96.31)	<0.0001
Household food insecurity (n = 67,550) *							
Percentage (95% CI)	7.1% (6.7–7.4)	14.8% (13.5–16.1)	<0.0001	14.5% (13.2–15.8)	<0.0001	52.5% (29.7–75.3)	<0.0001
AOR (95% CI)	1 (ref)	1.98 (1.74–2.26)	<0.0001	1.94 (1.70–2.22)	<0.0001	8.80 (3.53–21.95)	<0.0001

Abbreviations: AOR, adjusted odds ratio; CI, confidence interval. * Unweighted numbers were reported. They were rounded to base 50 for confidentiality purposes according to Statistics Canada data release policies. Percentages were based on weighted numbers, and have been adjusted by age and sex. † For all comparisons, people with no illicit drug use constituted the reference category.

**Table 5:** Association of prior illicit drug use with past-year suicidal behaviors, and past-year household food insecurity.

Association with Outcome	No Illicit Drug Use	Any Illicit Drug Use	*p* Value †	Any Illicit Drug Use Non-Injecting Drug Use	*p* Value †	Injecting Drug Use	*p* Value †
Prior illicit drug use							
Suicidal behaviors (n = 106,100) *							
Suicidal ideation							
Percentage (95% CI)	2.4% (2.2–2.5)	2.9% (2.5–3.2)	0.0058	2.8% (2.4–3.1)	0.0248	10.6% (2.8–18.4)	0.0002
AOR (95% CI)	1 (ref)	1.21 (1.04–1.40)	0.0108	1.17 (1.01–1.35)	0.0371	3.75 (1.55–9.05)	0.0033
PAF (95% CI)	··	16.6% (4.2–27.5)	··	13.9% (9.2–25.2)	··	70.3% (36.2–86.1)	··
Suicidal plans							
Percentage (95% CI)	0.7% (0.6–0.8)	0.96% (0.76–1.16)	0.0031	0.95% (0.75–1.15)	0.0048	2.0% (0.0–4.0)	0.0380
AOR (95% CI)	1 (ref)	1.48 (1.15–1.91)	0.0026	1.47 (1.13–1.90)	0.0036	2.07 (0.71–6.01)	0.1820 ‡
PAF (95% CI)	··	31.9% (12.6–47.0)	··	31.5% (11.7–46.8)	··	NC	··
Suicidal attempts							
Percentage (95% CI)	0.38% (0.30–0.45)	0.37% (0.25–0.49)	0.9057	0.37% (0.25–0.49)	0.9005	0.42% (0.00–1.15)	0.9044
AOR (95% CI)	1 (ref)	0.91 (0.60–1.38)	0.6531	0.91 (0.59–1.40)	0.6748	0.66 (0.11–3.85)	0.6473
PAF (95% CI)	··	NC	··	NC	··	NC	··
Household food insecurity (n = 67,550) *							
Percentage (95% CI)	7.9% (7.5–8.2)	8.2% (7.6–8.9)	0.3091	8.1% (7.5–8.8)	0.5255	19.7% (13.1–26.4)	<0.0001
AOR (95% CI)	1 (ref)	1.27 (1.14–1.41)	<0.0001	1.25 (1.12–1.40)	0.0001	2.25 (1.42–3.57)	0.0005
PAF (95% CI)	··	17.7% (10.0–24.8)	··	16.9% (9.1–24.1)	··	45.1% (24.6–60.0)	··

Abbreviations: AOR, adjusted odds ratio; CI, confidence interval; PAF, population attributable fraction; NC, not computed because the AOR was not significant. * Unweighted numbers were reported. They were rounded to base 50 for confidentiality purposes according to Statistics Canada data release policies. Percentages were based on weighted numbers, and have been adjusted by age and sex. † For all comparisons, people with no illicit drug use constituted the reference category. ‡ The non-significant *p* value was likely owing to an underpowered model.

## Data Availability

All data are available in the present manuscript.
